# Measurement of blood pressure in the leg—a statement on behalf of the British and Irish Hypertension Society

**DOI:** 10.1038/s41371-020-0325-5

**Published:** 2020-04-22

**Authors:** James P. Sheppard, Peter Lacy, Philip S. Lewis, Una Martin, on behalf of the Blood Pressure Measurement Working Party of the British and Irish Hypertension Society, N. Chapman, P. Chowienczyk, C. Clark, S. McDonagh, E. Denver, R. McManus, A. Neary

**Affiliations:** 1 Nuffield Department of Primary Care Health Sciences, University of Oxford, Oxford, UK; 2 Population Science & Experimental Medicine, Institute of Cardiovascular Science, University College London, London, UK; 3 Stockport NHS Foundation Trust, Stockport and University of Manchester, Manchester, UK; 4 School of Pharmacy, University of Birmingham, Birmingham, UK; 5 Imperial College London, London, UK; 6 King’s College London, London, UK; 7 University of Exeter, Exeter, UK; 8 Whittington Health NHS Trust, London, UK; 9 University of Oxford, Oxford, UK; 10 Galway Clinic, Galway, Ireland

**Keywords:** Diagnosis, Hypertension

## Executive summary

Ankle blood pressure (BP) measurement is necessary for the diagnosis of hypertension where measurements are not possible due to medical conditions or limb deformities. Based on a recent review of the evidence, we recommend an ankle BP threshold of ≥155/90 mmHg to define high blood pressure in patients who do not have vascular disease. We recommend that ankle BP readings are taken with the subject lying down, using a validated automated device with the cuff placed around the ankle/lower calf.

## Full statement

Blood pressure (BP) is normally measured on the upper arm, but occasionally this is not possible. The presence of fractures, wounds, vascular access devices and shunts, morbid obesity, surgical procedures, lymphoedema, limb deformities (phocomelia) and amputations may prevent the satisfactory cuff placement around the upper arm. In addition, BP measurement may be inaccurate in the presence of bilateral subclavian artery stenoses such as can occur with Takayasu’s arteritis [1] or atherosclerosis [2]. In these circumstances, measurement of BP in the leg may be necessary. It is important to recognise, however, that BP measurements in the arm may differ from those in the legs.

A recent systematic review examined the relationship between supine BP measurements in the arm and leg [3]. A review of 44 studies involving 9771 patients concluded that ankle systolic BP was on average 17.0 mmHg (95% CI 15.4–21.3 mmHg) higher than arm systolic BP, whilst there was no difference in diastolic BP in the general population [3]. These findings suggest that a threshold of ≥155/90 mmHg could be used for diagnosing hypertension in routine practice when only ankle measurements are available. This threshold is conservative and would ensure maximum sensitivity to detect hypertension at the expense of some specificity.

It should be noted that the review found much lower leg pressures in the presence of peripheral vascular disease (PVD). The proposed threshold should therefore be used with caution, and patients with low ankle BPs in the presence of cardiovascular risk factors (e.g. diabetes, renal disease and existing cardiovascular disease) should be considered for further investigation, especially if there is a history of intermittent claudication or clinical evidence of PVD (e.g. femoral arterial bruits, poor or absent foot pulses, poor distal skin perfusion, cold peripheries or arterial ulceration). In such cases, arterial Doppler ultrasonography, CT or MR angiography can be used to confirm significant PVD which may invalidate the use of ankle BP as a surrogate for arm BP. PVD may be worse in one leg compared with the other, so ankle BPs should be taken in both legs where this is suspected.

**Fig. 1:**
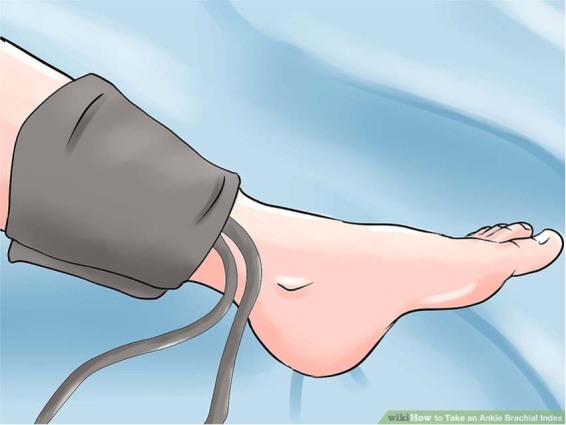
Positioning of the blood pressure monitor cuff on the ankle/lower calf. Image taken from Wiki How to take an Ankle Brachial Index (https://www.wikihow.com/Take-an-Ankle-Brachial-Index).

The review found no consistent or accepted method for measuring BP in the leg. We therefore propose that ankle BP is measured in a supine position, using a cuff placed around the ankle/lower calf (Fig. 1), ensuring the bladder encircles ≥80% of the ankle circumference. Readings should be taken either by oscillometry or Doppler readings of return to flow at the dorsalis pedis or posterior tibial arteries (systolic readings only). Auscultation is not feasible in most subjects and is not therefore recommended. Ankle BPs are recommended rather than calf or thigh measurements because they generally cause less discomfort and the cuff is easier to fit, particularly in obese patients. As with standard clinic BP measurement, readings should be taken after a 5-min rest period [4]. In terms of oscillometric BP monitors, these have not been specifically validated for leg measurements but are widely used in clinical practice and are a reasonable choice. It is important to note that the use of ambulatory readings for diagnosis will not be possible in patients requiring leg BP measurements. However, where out-of-office measurements are required, home ankle BP monitoring could be considered after appropriate training.

Ankle BP measurement represents a viable alternative to arm measurement for the diagnosis of hypertension, where placement of a cuff on the upper arm is not possible. A threshold of ≥155/90 mmHg can be recommended, but physicians should use it with caution, recognising that ankle BP measurements may differ significantly in patients with PVD. Given the impracticalities of taking ambulatory measurements in the ankle, we recommend that diagnosis is confirmed and treatment initiated only following consistently high ankle BP readings from repeated clinic visits.
